# Successful Outcomes following Repair of a Type A Aortic Dissection during Pregnancy

**DOI:** 10.1055/s-0040-1701605

**Published:** 2020-06-29

**Authors:** Akbarshakh Akhmerov, Nicola D'Attellis, Aamir S. Shah

**Affiliations:** 1 Department of Surgery, Cedars-Sinai Medical Center, Los Angeles, California; 2 Department of Cardiac Surgery, Smidt Heart Institute, Cedars-Sinai Medical Center, Los Angeles, California; 3 Department of Anesthesiology, Cedars-Sinai Medical Center, Los Angeles, California; 4 Department of Cardiac Surgery, Cedars-Sinai Medical Center, Los Angeles, California

**Keywords:** aortic dissection, aorta, pregnancy, thoracic, multidisciplinary

## Abstract

Type A aortic dissections during pregnancy are rare but lethal events, with reported mortalities as high as 60%. Unique changes in hemodynamics, hormone-related alterations in aortic tissue, and preexisting risk factors place patients at an elevated risk for dissection. We report a successful repair of a Type A aortic dissection at 32 weeks of gestation, with excellent outcomes in both mother and child. This report highlights the importance of gestational age and multidisciplinary effort.

## Introduction

Type A aortic dissections are a rare but lethal entity in the pregnant population. The incidence varies from 0.4 to 3.5 cases per 100,000 person-years, and the overall mortality can be as high as 60%. 1 2 Dissections typically occur in the third trimester, concurrent with hemodynamic stressors (e.g., increased stroke volume, heart rate, and cardiac output). 3 Operative mortality for both mother and fetus varies widely, depending on the sequence of operations and gestational age—this underlines the importance of a multidisciplinary approach. We report a case of a successful cesarean delivery followed by repair of a Type A aortic dissection using central cannulation in a 32-week gestational pregnancy.

## Case Presentation

**Fig. 1:**
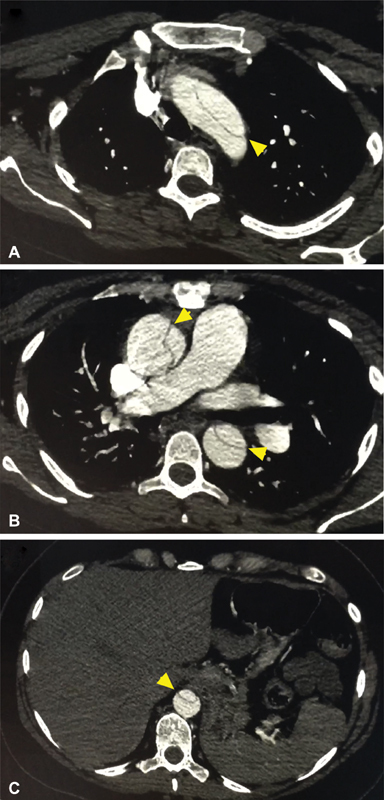
Preoperative images of the aortic dissection at the level of the aortic arch ( **A** ), main pulmonary artery ( **B** ), and celiac trunk ( **C** ).

A 32-year-old female with a 32-week gestational pregnancy (gravida 4, para 1) presented to an outside facility with shortness of breath and acute chest pain, radiating to the back and abdomen. Her prenatal course was complicated by gestational hypertension, for which she was not taking antihypertensive medications. There was no personal or family history of connective tissue disorders. A computed tomography angiogram of the chest, abdomen, and pelvis revealed a Type A dissection extending from the aortic valve plane to the bilateral external iliac arteries ( Fig. 1 ). The aorta was dilated to 48 mm above the valve plane. Although the dissection flap extended into the origins of the innominate, left common carotid, and left subclavian arteries, there was no progression beyond the origins. The celiac, mesenteric, and renal arteries were patent, without evidence of malperfusion. After receiving a single dose of betamethasone the patient was transferred to our facility for definitive therapy. En route, esmolol infusion was initiated to maintain a systolic blood pressures below 120 mm Hg. Care was taken, however, to prevent hypotension that could compromise fetal perfusion. Fetal heart rate monitoring was used to assess the well-being of the fetus. Upon arrival to our facility, the patient was emergently taken to the operating room, with cardiac and obstetric anesthesiology, cardiothoracic surgery, high-risk obstetrics, and neonatology teams present.

In the operating room the patient and fetus were immediately assessed by a joint, interdisciplinary cardiac–obstetric anesthesia team. Left uterine displacement was ensured, and the abdomen was prepped and draped for an emergent cesarean section prior to induction of general anesthesia. A rapid sequence induction using propofol, fentanyl, and succinylcholine was performed. General anesthesia was maintained with sevoflurane (0.7–1.0%) and bolus infusions of fentanyl. An 8-French quadruple lumen and a 9.5-French sheath introducer were placed in the right internal jugular vein.

A low transverse cesarean section was performed and the baby was delivered from a transverse presentation. After closure of the hysterotomy, the abdomen was left open to monitor for intraoperative uterine bleeding while the aortic repair was in progress. The patient received two infusions of oxytocin (40 units each) prior to full heparinization and cardiopulmonary bypass (CPB), and an additional dose during CPB. Oxytocin infusion was maintained for 24 hours. The anesthesia team adjusted its choice and dosage of anesthetics to minimize neonatal depression. Upon delivery, however, the neonate exhibited hypotonia and received mechanical ventilation for respiratory support.

**Fig. 2:**
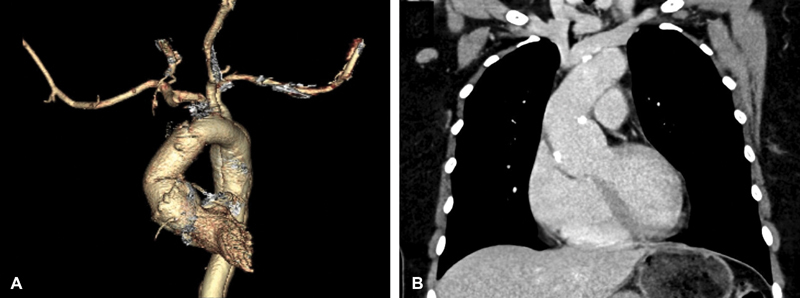
Postoperative follow-up showing successful repair of a Type A aortic dissection on a 3D reconstructed image ( **A** ) and plain computed tomography ( **B** ). 3D, three dimensional.

The aortic repair commenced after the cesarean section. Intraoperative transesophageal echocardiography (TEE) demonstrated normal biventricular function, a tricuspid aortic valve with mild insufficiency, and sinus of Valsalva diameter of 38 mm. The femoral vessels were deemed unsuitable for arterial cannulation due to size and possible involvement of the dissection. Therefore, central aortic cannulation was utilized. Direct cannulation of the ascending aorta along the lesser curve was accomplished using a Seldinger technique, with TEE confirmation of the guidewire within the true lumen. A dual-stage venous cannula was introduced into the right atrium via the atrial appendage and CPB was established. A retrograde cardioplegia catheter and a superior vena cava (SVC) cannula for retrograde cerebral perfusion were placed. With systemic temperature of 18°C, deep hypothermic circulatory arrest was initiated. Retrograde cerebral perfusion via the SVC cannula was established at 10 mL/kg/min. Upon direct inspection of the aorta, the intimal tear was noted to be at the sinotubular junction (STJ), with slight extension into the noncoronary sinus. The sinuses were not significantly dilated and the aortic valve demonstrated normal leaflet coaptation. The repair was performed with replacement of the ascending aorta and hemiarch from the STJ, with resuspension of aortic valve commissures. A 28-mm Gelweave graft (Vascutek Ltd, Scotland, UK) was used for the repair. Total circulatory arrest time was 30 minutes. Following repair, intraoperative TEE revealed a normal aortic valve and biventricular function. The hysterotomy was again inspected and appeared hemostatic. The abdomen was closed by the obstetrics team and the patient was transported to the intensive care unit for close observation. Postoperatively, the patient's blood pressure and heart rate were maintained at ≤120 mm Hg and 60 beats/min, respectively. She was extubated on postoperative day zero, and, after an unremarkable recovery, discharged on postoperative day eight. Similarly, the neonate made an unremarkable recovery. One-year follow-up imaging demonstrated a stable aortic repair ( Fig. 2 ).

## Discussion

Aortic dissections during pregnancy typically originate in the thoracic aorta, with 89% arising in the ascending aorta. 4 The majority of dissections occur during the third trimester, which coincides with important hemodynamic changes, including increase in heart rate, cardiac output, stroke volume, and compression of the distal aorta and iliac vessels by the gravid uterus. Hormonal factors may also play a role. For example, estrogen receptors have been identified in aortic tissue, and hormonal influence may lead to loss of normal architecture of elastic fibers. 3 5 Hypertensive complications of pregnancy, like preeclampsia, also confer a risk. 6

Thus, it is not surprising that pregnancy has been proposed as an independent risk factor for aortic dissection. More recently, however, the association between pregnancy and aortic dissection has been attributed to selective reporting. In a longitudinal, population-based study, pregnancy and aortic dissections were shown to be nonrelated events (relative risk: 3.27, 95% confidence interval: 0.82–12.95; *p* = 0.14). 2 Although pregnancy may not be a risk factor per se, certain characteristics during pregnancy place patients at an increased risk. These include hypertension, connective tissue disorders, aortic root enlargement (>4 cm), or an increase in aortic root size during pregnancy in patients with bicuspid aortic valves. 3 7 Therefore, consideration of all potential risk factors is important.

The sequence of operations (i.e., cesarean section followed by aortic repair vs. aortic repair with the fetus in situ and subsequent delivery) is essential for successful outcome. The primary objective is survival of two lives, and gestational age figures prominently into this objective. Zeebregts et al 9 recommend aortic repair with the fetus in situ before 28 weeks of gestation, and primary cesarean section followed by aortic repair after 32 weeks of gestation. Between 28 and 32 weeks of gestation, the approach is dictated by the condition of the fetus, although Zhu et al 8 demonstrate adequate survival rates with delivery-first approach after 28 gestational weeks. If there are signs of fetal distress, immediate delivery is recommended. Otherwise, pregnancy should be prolonged. 9

To determine the optimal sequence and conduct of the operation, a multidisciplinary approach is necessary. The surgical teams (cardiac and obstetric) in this case coordinated the sequence of the operations and discussed management of the abdomen after delivery of the child. Specifically, the abdomen remained open during the aortic repair, allowing the team to monitor the hysterotomy for hemorrhage. A contingency plan for hysterectomy was also discussed, in the event of hemorrhagic shock. Neonatal intensivists and anesthesiologists played equally important roles in ensuring timely intervention and resuscitation.

The postoperative course is also a multidisciplinary effort. Although the patient was primarily managed in the cardiac intensive care unit, she was closely followed by the obstetrics team and received postoperative infusion of oxytocin. Similarly, the patient's child recovered in the neonatal intensive care unit, where he was successfully weaned from mechanical respiratory support.

Type A aortic dissections during pregnancy are rare events but carry an elevated risk of mortality for both mother and fetus. Consideration of gestational age is of paramount importance, and early multidisciplinary coordination is mandatory for a successful outcome. Here, we report a successful delivery of a child at 32 weeks of gestation, followed by repair of a Type A aortic dissection. Follow-up at 2 years demonstrates no adverse sequelae in either mother or child.
